# Quick self-assembly of bio-inspired multi-dimensional well-ordered structures induced by ultrasonic wave energy

**DOI:** 10.1371/journal.pone.0246453

**Published:** 2021-02-24

**Authors:** Connor Murphy, Yunqi Cao, Nelson Sepúlveda, Wei Li

**Affiliations:** 1 Department of Mechanical Engineering, University of Vermont, Burlington, Vermont, United States of America; 2 College of Control Science and Engineering, Zhejiang University, Hangzhou, Zhejiang, China; 3 Department of Electrical and Computer Engineering, Michigan State University, East Lansing, Michigan, United States of America; University of Louisville, UNITED STATES

## Abstract

Bottom-up self-assembly of components, inspired by hierarchically self-regulating aggregation of small subunits observed in nature, provides a strategy for constructing two- or three-dimensional intriguing biomimetic materials via the spontaneous combination of discrete building blocks. Herein, we report the methods of ultrasonic wave energy-assisted, fast, two- and three-dimensional mesoscale well-ordered self-assembly of microfabricated building blocks (100 μm in size). Mechanical vibration energy-driven self-assembly of microplatelets at the water-air interface of inverted water droplets is demonstrated, and the real-time formation process of the patterned structure is dynamically explored. 40 kHz ultrasonic wave is transferred into microplatelets suspended in a water environment to drive the self-assembly of predesigned well-ordered structures. Two-dimensional self-assembly of microplatelets inside the water phase with a large patterned area is achieved. Stable three-dimensional multi-layered self-assembled structures are quickly formed at the air-water interface. These demonstrations aim to open distinctive and effective ways for new two-dimensional surface coating technology with autonomous organization strategy, and three-dimensional complex hierarchical architectures built by the bottom-up method and commonly found in nature (such as nacre, bone or enamel, etc.).

## Introduction

Spontaneous organization of the building elements into well-ordered patterns or structures is of long standing and continuing interest for its aesthetic appeal, and for potential as a promising strategy to mimic the formation of useful and intriguing structures that exist in nature [1–6]. Driven by the attribute of a system to minimize its energy by spontaneous assembly of individuals units, self-assembly provides an effective route to well-defined structures that are close, or at a thermodynamic equilibrium state. Covalent synthesis is accomplished and is successful for making numerous target molecules. Self-assembly is key to noncovalent synthesis which enables creation of large aggregates of possible structured matter with structural complexity such as macromolecules or virus particles that are hard to realize by covalent synthesis [7–10]. In a typical self-assembling process, predesigned building blocks spontaneously organize themselves into a relatively stable structure through non-covalent interactions [11–13].

Self-assembling process could be categorized by the size of the building blocks of nanoscale self-assembly and mesoscale self-assembly, provided that appropriate conditions are met [11, 14]. The focus on self-assembly as a strategy for synthesis has been confined largely to molecules and nanoscales, because the chemists and material scientists are professionally concerned with manipulating the material structure at these scales [7, 15–17]. Many of the studies concerning self-assembly of small objects have dealt mainly with colloidal crystals-ordered arrays of particles having dimensions ranging from 1 nm to 100 nm [18–20]. Examples of colloidal crystal formation in natural systems include the geological production of opals and the biological crystallization of proteins and viruses [21, 22]. Chemically synthesized colloidal particles with a spherical shape and size ranging from a few nanometre to a few micrometre have been actively explored [11]. DNA has also been used as a building block to enable self-assembly into two- and three-dimensional structures [23–25]. Self-assembling processes are mainly focused on the understanding and development of a building process based on molecules and nanoparticles [26]. It is also intriguing to explore methods and techniques regarding self-assembly of relatively large objects with mesoscopic scale (several orders larger than that of molecular level self-assembly), particularly due to the problems with stability of the system. Well-ordered self-assembly of mesoscale building blocks requires much stronger surface attractive force to yield patterned and stable structures to overcome much larger destructive inertia forces. Due to these challenges, few studies on self-assembly of well-ordered stable structures made of building blocks with dimensions larger than 10 μm have been reported [21, 27, 28]. Technologies based on mesoscale self-assembly have emerged as a promising approach for the autonomous construction of the predesigned pattern into macroscopic size [7, 29, 30]. Not only do the characteristic dimensions of mesoscopic building blocks approximate to that of structural units arranged in complex hierarchical architectures of natural material, but also the increased feature size would enable building blocks with more complex and varied geometries to be mass-produced by using microfabrication process. Moreover, compared to nanoscale self-assembly, the dimensional gap between the fundamental building elements and the targeted macroscopic functional structure has narrowed significantly, resulting in macroscopic bioinspired structural material that mimic their natural counterpart in regard to geometry and bottom-up formation.

Although much of the work in self-assembly has focused on molecular components, many intriguing applications of self-assembly strategy can be found at larger sizes [7]. Self-assembly of components larger than molecular level into ordered arrays is an efficient way of preparing structural materials with interesting mechanical, electrical and optical properties [31]. For example, the potential for generating complex internal shapes make them attractive candidate materials with photonic band gap properties, as components of heat exchangers, as support for catalysis or chromatography, microelectronics, MEMS, sensors and micro-analytical devices [32–35]. In industrial applications, one example is that fluidic self-assembly is capable of patterning micromachined silicon parts onto silicon and quartz substrates in a preconfigured design [34]. Additionally, self-assembled GaAs/GaAlAs LEDs with electrical connections have been demonstrated [30, 35]. In order to create large-scale highly ordered structures with self-assembly, a variety of mechanisms have been employed, including magnetic attraction [3, 36], capillary interaction [11, 27, 37], electrostatic force [38, 39], long-range dipolar attraction [40], etc. In nature, the formation of complex structure through self-regulating aggregation of periodic elements is a broadly observed strategy [37]. Mesoscale self-assembly is also promising to duplicate the interesting structure of natural material, such as mineralized tissues of vertebrates (bone, teeth, and calcified tendons), as well as the outer skeleton of invertebrates [41]. Nacre may serve as an example for natural nano-composite with its high toughness and a hierarchical brick-mortar structure consisting of mineral tablets [33, 42]. Chemical synthesized colloidal particles are typically characterized by a simple spherical shape and nanoscale size. However, microfabrication by photolithography is broadly generalized into a variety of shapes, sizes, and materials, and can generate large numbers of indistinguishable devices. Using this method, building blocks for self-assembly with special geometry sizing on the order of hundreds of nanometers to hundreds of micrometers can be attained, whereas nanoscale building blocks are mostly restricted to spheres, nanorods, or cubes [27]. Mesoscale self-assembly has the potential to overcome some of the main limitations of molecular/nano-scale self-assembly. Building synthetic versions of sophisticated naturally occurring structural materials will likely benefit from innovative microfabrication and self-assembly method.

## Materials and methods

### Fabrication of hexagonal polysilicon microplatelets

A 6-inch silicon-on-insulator wafer (Ultrasil Corporation) with device layer thickness of 30 +/− 1 μm and buried thermal oxide thickness of 2 μm +/− 5% was first dehydrated and cleaned by O_2_ plasma (Matrix asher Model 106). The wafer was then spin-coated 1.2 μm thick i-line positive photoresist and soft baked (Picotrack Spin Coat Track). Next, the mask pattern of microplatelets was transferred to the wafer by photolithography (GCA-8500 wafer stepper) capable of resolving sub-micron features. After the exposed wafer was developed (Picotrack Spin Develop Track), a photostabilizer system (Axcelis M200PCU) used both ultraviolet light and a heated chuck respectively to cross-link and harden patterned photoresist. The shapes of hexagonal microplatelets were defined by using deep reactive-ion etching (DRIE) in an inductively coupled plasma etch system (STS Advanced Silicon Etcher). After the photoresist was removed by plasma asher, the wafer was immersed in 49% aqueous hydrofluoric acid in a Teflon beaker for 3 hours. Since there was a strong hydrophobic interaction between the released microplatelets and handle wafer, the wafer was then transferred to a clean glass beaker filled with deionized water, followed by putting the glass beaker into an ultrasonic bath (Branson 5510) for 2 hours. After microplatelets were fully detached from the handle wafer, a vacuum filtration system was used to collect them from the deionized water in the glass beaker. Finally, all collected microplatelets were cleaned in a volume ratio 4:1 solution of concentrated H_2_SO_4_: 30% H_2_O_2_ for 20 min and rinsed in deionized water prior to surface-functionalization.

### Surface-functionalization for water-air interface self-assembly

Prior to surface-functionalization, 1 mL of 3-aminopropyltriethoxysilane (ATES, Sigma-Aldrich) was mixed for 1 hour in 10 mL of a water-methanol solution with volume ratio of 3:1 in order to fully hydrolyze the silane species. Surface-functionalization was then accomplished by adding 0.14 g of microplatelets into the hydrolysed ATES solution, followed by stirring for 1 hour at room temperature. Then, the modified microplatelets were washed 2 times by centrifugation with pure ethanol, followed by washing 2 times with deionized water. Finally, the surface-functionalized microplatelets were stored as a stock suspension in deionized water in a plastic tube.

### Water-air interface self-assembly inside water droplet

Droplets of modified microplatelets suspension were transferred onto the inner surface of 2-inch plastic Petri dish (VWR, LLC) through a pipette. Then, the Petri dish was quickly flipped upside down to make the droplets hang onto the inner surface of Petri dish without dropping due to the balance of surface tension and gravity. The self-assembling process of microplatelets inside the droplets was examined by an inverted microscope (Nikon Eclipse Ti). Mechanical impulses were applied by gentle shocks on top surface of the Petri dish. The resulting vibration propagated from the Petri dish to the hanging droplet and finally agitated the functionalized microplatelets to facilitate the self-assembly. The process took less than 1 minute to form an ordered and monolithic self-assembled structure.

### Surface-functionalization for multi-dimensional self-assembly

200 μL of Trichloro(1H,1H,2H,2H-heptadecafluorodecyl)silane (TCI America, Inc.) was first mixed with 10 ml ethanol for 1 hour. Surface modification was then accomplished by adding microplatelets into the mixed solution, followed by stirring for 24 hours at room temperature. The modified microplatelets were then washed 2 times by repeated centrifugation with pure ethanol and stored as a stock suspension consisting of 10 mL pure ethanol. Next, 300 μL of n-dodecyl methacrylate (Sigma-Aldrich) was added into suspension, followed by ultrasonication for 5 minutes, and stirring at room temperature for 1 hour to fully dissolve the dodecyl methacrylate. The microplatelets were then washed 2 times by repeated centrifugation with pure ethanol.

### Multi-dimensional self-assembly inside water and at water-air interface

Prior to self-assembly, the fully surface-functionalized microplatelets was stored in a glass vial filled with pure ethanol. After microplatelets were settling down to the bottom of glass vial, a pipet was carefully used to withdrew nearly all the ethanol/dodecyl methacrylate solution, leaving enough to barely cover the microplatelets. Then, deionized water was continuously added to the microplatelets at the bottom of glass tube to make dodecyl methacrylate stay around the functionalized faces of microplatelets, followed by rinsing away the excess ethanol and dodecyl methacrylate. After filling with 10 mL deionized water, unordered aggregation of microplatelets emerged immediately (S1 Fig in S1 File). Next, the glass tube with the microplatelets at the bottom was transferred into an ultrasonic bath with the operating frequency of 40 kHz for 30 minutes. The experimental results of two-dimensional and three-dimensional self-assembly in the glass tube were examined by a stereomicroscope (Nikon SMZ 1500).

## Results and discussion

### Preparation of building blocks for self-assembly

**Fig 1:**
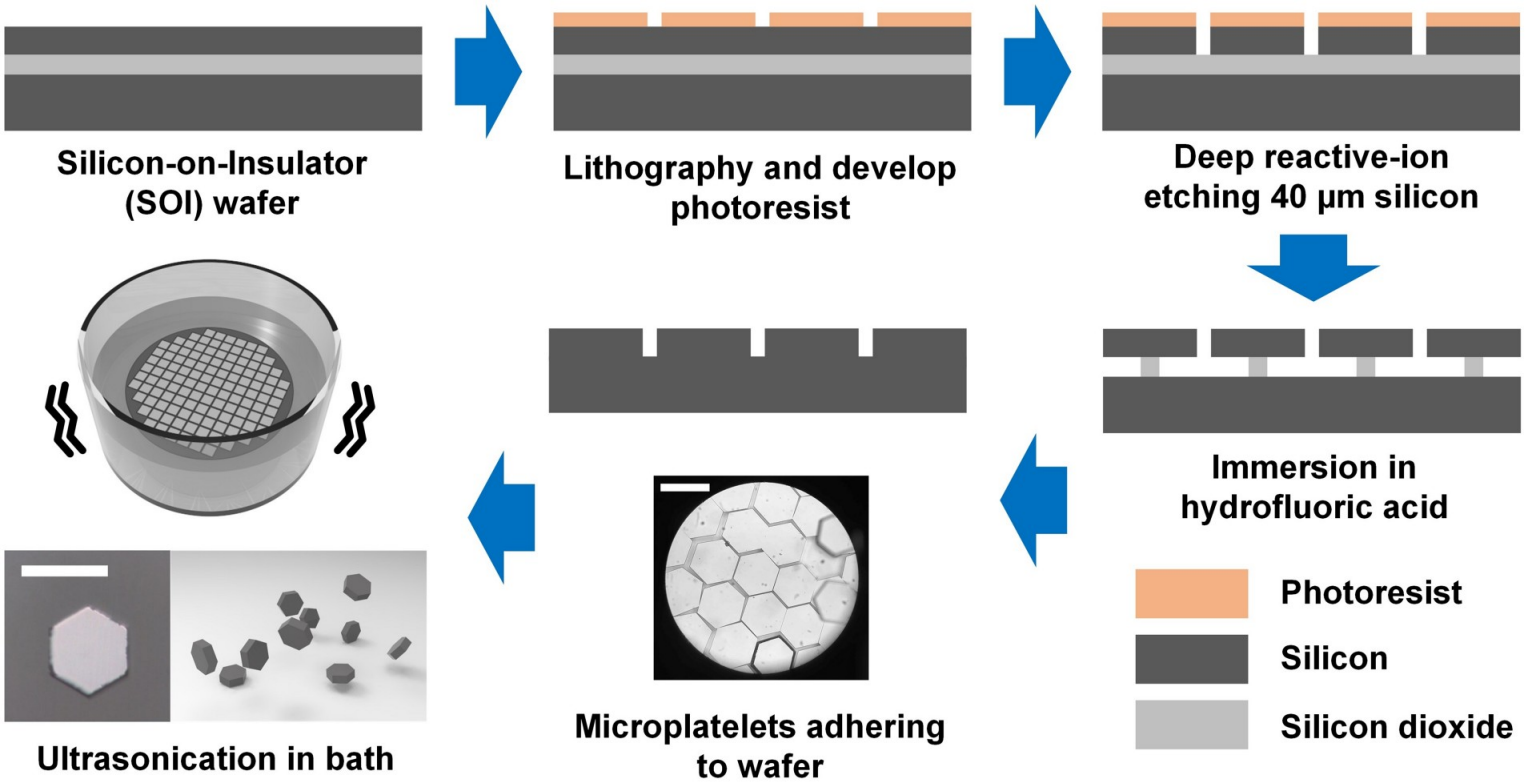
Fabrication and releasing of the hexagonal microplatelets made of polysilicon as the building blocks for 100-μm-size mesoscale self-assembly (scale bars are 100 μm).

In order to promote the self-assembly of mesoscale subunits into highly periodic, space filling two- and three-dimensional structures, we designed and microfabricated precise uniform hexagonal microplatelets with thickness of 30 μm and width of 100 μm. The volume of individual mesoscale building block in this study is about 187 times that of the building block of previous remarkable mesoscale self-assembly research [27, 28]. A much larger volume of building blocks would produce much greater destructive inertia force during the self-assembling process. Fig 1 summarizes the primary fabrication steps for the hexagonal microplatelets made of polysilicon as the building blocks for mesoscale self-assembly. The predesigned microplatelets were fabricated from 6-inch silicon-on-insulator (SOI) wafers with silicon layer thicknesses of 30 μm and buried thermal oxide thicknesses of 2 μm. The hexagonal shape of microplatelets were defined using DRIE with patterned i-line photoresist as masking layer. After etching the sacrificial silicon dioxide layer for releasing the microplatelets, hydrofluoric acid rendered both surfaces of polysilicon microplatelets and silicon handle wafer hydrophobic. Due to this hydrophobic interaction between silicon surfaces, the microplatelets were released, but still firmly adhered to the silicon handle wafer after the oxide in between was completely etched. This attractive interaction was so strong that rinsing the wafer with fresh methanol was not able to detach microplatelets from the handle wafer. To fully release and collect the polysilicon microplatelets, the wafer with microplatelets on top was transferred into a clean glass beaker filled with deionized water; then the glass beaker was placed in an ultrasonic bath for 2 hours. The mechanical energy generated from the sonication bath agitated the microplatelets, making them fully detached from the handle wafer, and subsequently collected by filtration.

### Self-assembled three-dimensional “soccer balls”

**Fig 2:**
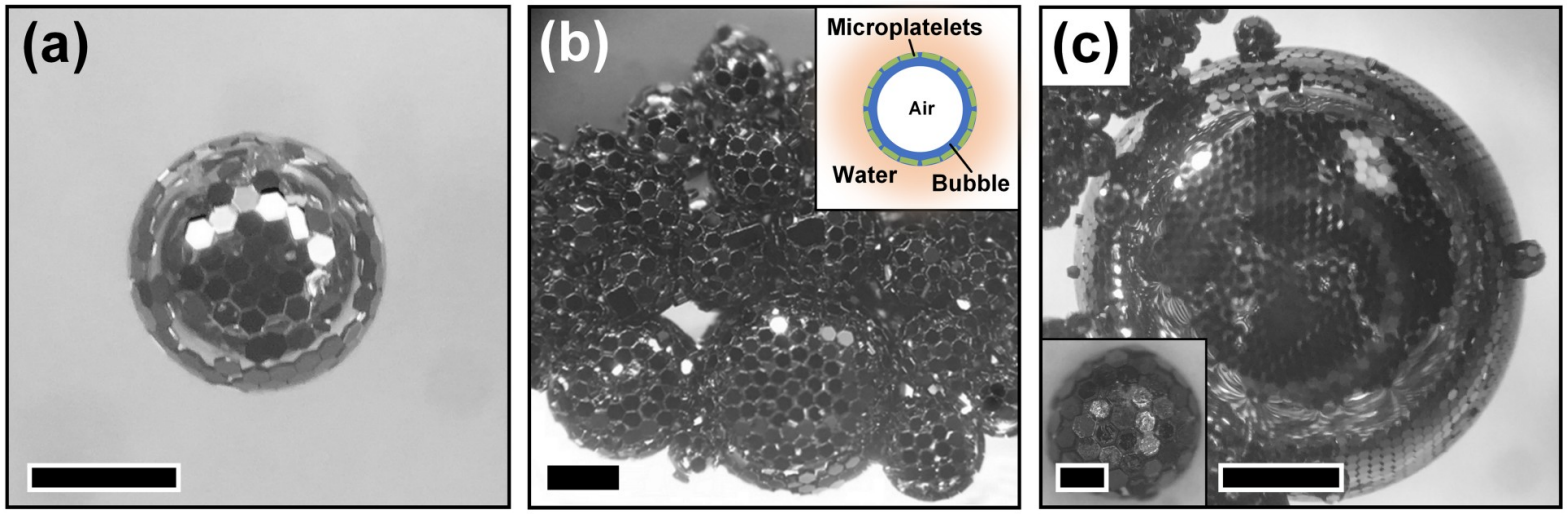
Optical images of self-assembled three-dimensional “soccer balls” by hexagonal polysilicon microplatelets at the water surface in the glass beaker. (a) An individual “soccer ball” showing the organized pattern of microplatelets (scale bar is 500 μm). (b) A cluster of “soccer balls” randomly generated bubbles by ultrasonic waves and the schematic illustration of the “soccer ball” (inset; scale bar is 400 μm). (c) The “soccer balls” varying in size associated with the size of the bubbles with smaller “soccer ball” with the diameter about 700 μm (inset; scale bar is 200 μm) and larger “soccer ball” with diameter about 4 mm (scale bar is 1 mm).

During the releasing process of polysilicon microplatelets, hydrofluoric acid removed both hydrophilically buried thermal oxide and oxide covered on the microplatelets due to the exposure to air and water. This process made all faces of polysilicon microplatelets to render hydrophobicity. High frequency vibration generated by ultrasonic bath propagated into the glass beaker, which was filled with deionized water and contained the handle wafer laying at the bottom with microplatelets sticking to it. The ultrasonic waves produced enough energy to shake the microplatelets off the handle wafer and forced them to vibrate in the water. Meanwhile, the ultrasonic waves created bubbles inside the water and bubbles continuously drifted up to the surface. Since these bubbles created the ideal water-air interfaces, hydrophobic microplatelets near the bubbles had a tendency of aggregating together to occupy these water-air interfaces in order to minimize their interfacial surface energy. Due to the massive weight of the individual building block, hydrophobic interaction was supposed to play a dominant role in the self-assembly over other fusion mechanisms that may occur at the nanometer scale or below. This phenomenon eventually enabled hexagonal microplatelets to self-assemble into stable three-dimensional “soccer balls”, and simultaneously drifted up to the surface of the water along with the bubbles (Fig 2a). It was observed that these self-assembled three-dimensional “soccer balls” varied in size (Fig 2b), which was associated with the size of the randomly generated bubbles by ultrasonic waves. Some three-dimensional “soccer balls” formed by self-assembly of hexagonal microplatelets can reach the size of about 4 mm in diameter determined by the bubble size inside (Fig 2c).

### Observation of monolayer self-assembly inside inverted droplets

**Fig 3:**
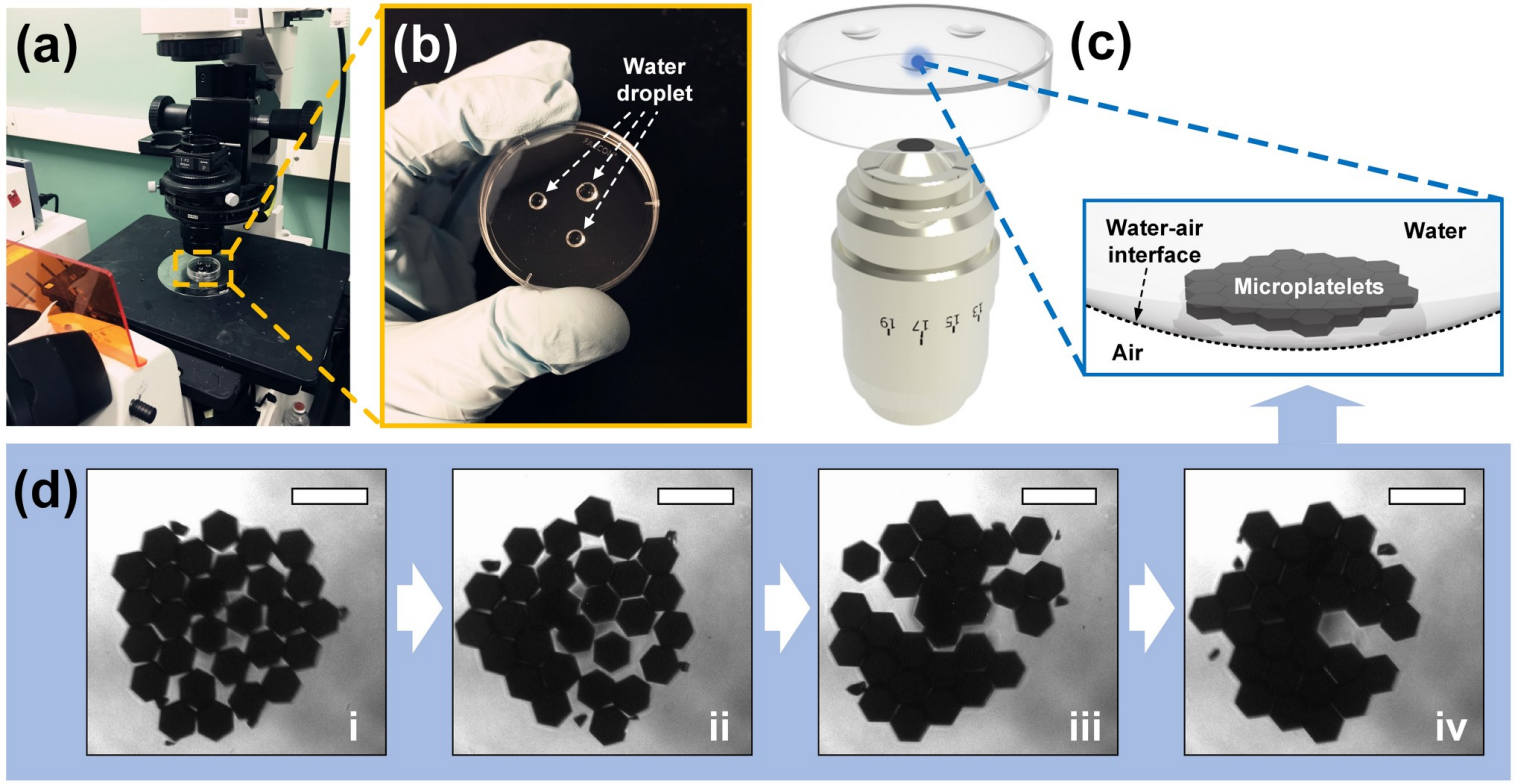
Monolayer mesoscale self-assembly of hexagonal microplatelets at water-air interface of droplets and real-time dynamical observation of the self-assembling process driven by mechanical agitation. (a) Experimental setup used for self-assembly, including an inverted plastic Petri dish and an optical microscope with inverted lens. (b) Three droplets of microplatelets suspension on the inner surface of a plastic Petri dish. (c) Schematic illustration of self-assembly of microplatelets at water-air interface of the droplets hanging at the bottom surface of the plastic Petri dish for inverted lens to observe. (d) Optical image frames of self-assembling process, showing the microplatelets gradually formed an integrated and well-ordered pattern (scale bar is 200 μm).

In order to use stronger hydrophobic interaction to drive the self-assembly of microplatelets and dynamically explore the real-time formation process of the ordered structure from discrete building blocks, faces of fabricated microplatelets were functionalized with ATES which was used as self-assembled monolayer by Bonderer et al. [41]. After surface-functionalization, all eight faces of the hexagonal microplatelets became hydrophobic, instigating a tendency to aggregate after being surrounded with water. The size of these microplatelets were large and heavy enough that the effects of gravity prevailed over the Brownian motion. Polysilicon microplatelets slowly sank into water body and eventually sedimented at the bottom of the container. The microplatelets that were surface-functionalized with ATES monolayer coating were discrete in water without forming a stable and ordered aggregation due to the fact that attractive surface interactions between polysilicon building blocks with large mass must overcome the strong inertia force. Since functionalized microplatelets with hydrophobic surfaces tended to move towards water-air interfaces in order to minimize their interfacial surface energy, we proposed a method to achieve the real-time dynamical observation of monolayer mesoscale self-assembly at the water-air interface driven by mechanical impulse (Fig 3 and S1 Movie). Microplatelets suspended in water droplets were hanging to the lower surface of a plastic Petri dish, and an optical microscope with inverted lens was used for observation. Gravitational force drove the microplatelets to the water-air interface of the droplet while the surface tension of water still kept them inside the droplets without falling off, creating an ideal scenario for real-time dynamical observation of microplatelets self-assembled at the bottom of the concave of droplet from a disorder state. Manually inputted mechanical impulses were discretely applied by using a tweezer to gently tap the Petri dish, causing the vibration to propagate from the Petri dish to the droplets holding the microplatelets. Due to the energy input, induced collisions between hexagonal microplatelets oriented and juxtaposed the hydrophobic faces of the adjacent microplatelets. In this case, discretely applying mechanical impulses with appropriate amplitude into the droplets minimized the defect of the self-assembly. The microplatelets were quickly self-assembling into small islands in the monolayer with an organized pattern. After the coalescence of these islands, an integrally and continuously self-assembled structure with well-ordered pattern and little defects were constructed by hexagonal microplatelets. It is worth noting that minimization of the defects in this self-assembled structure required the input energy to be within an appropriate range—strong agitation would result in the breakdown of already ordered pattern, whereas too weak agitation would fail to provide enough energy for the correction of the misalignment between individual building blocks.

### Organized surface coating by quick two-dimensional self-assembly

**Fig 4:**
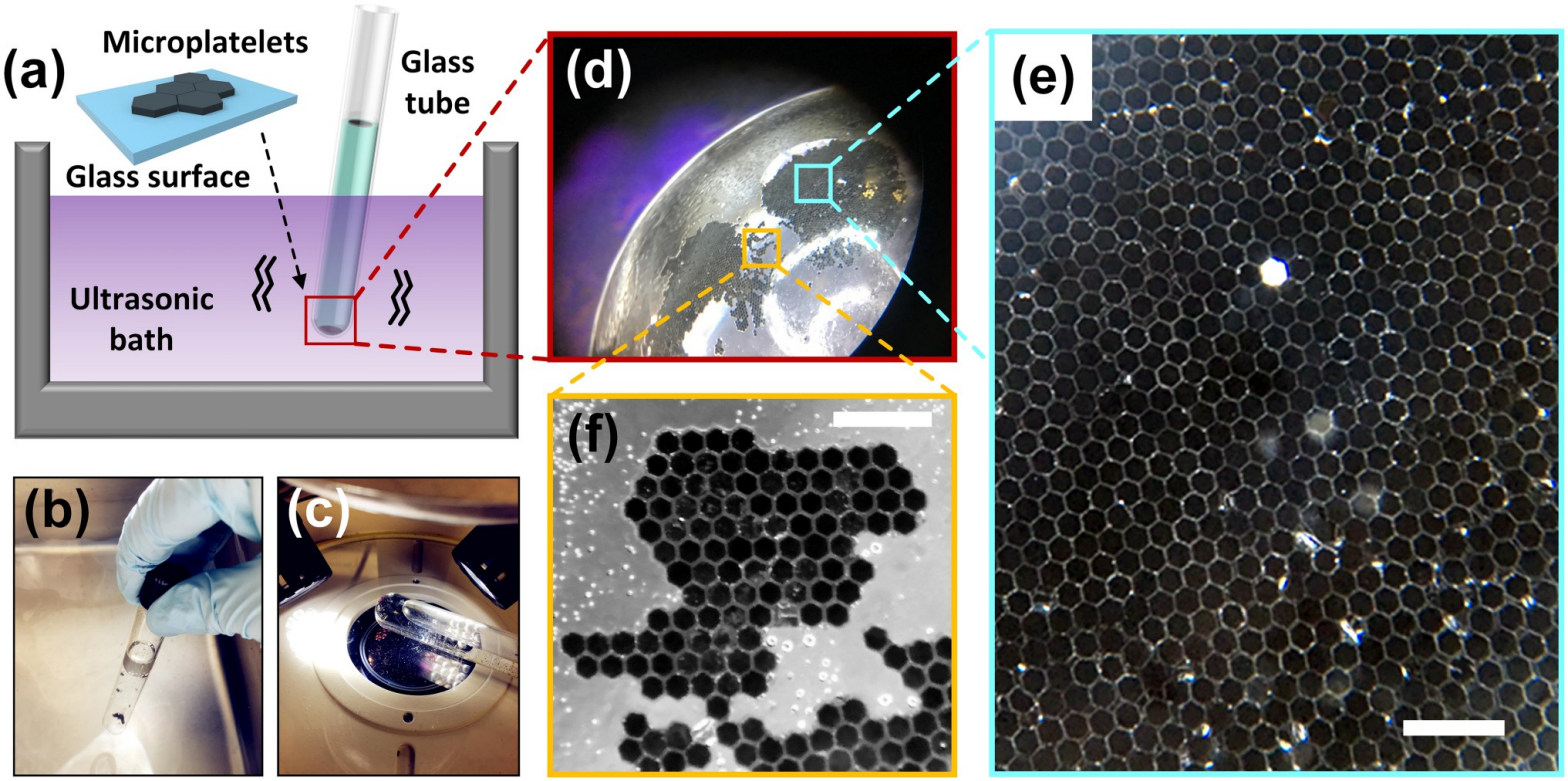
Highly ordered two-dimensional self-assembly of mesoscale hexagonal microplatelets in water. (a) Schematic illustration of the self-assembling process by using ultrasonic waves. Microplatelets self-assembled with top or bottom face adhering to the surface of the glass tube. (b-c) Photographs of (b) manipulation of self-assembly happening inside the glass tube by using ultrasonic waves, and (c) observation of organized pattern after self-assembly was finished by using stereomicroscope. (d) Optical image of the large area two-dimensional self-assembled aggregation at the bottom concave surface of the glass tube. (e-f) Zoom-in optical images of (e) area of dense and well-packed two-dimensional pattern formed by self-assembly (scale bar is 400 μm), and (f) defective area where self-assembled microplatelets still demonstrated precise organization as predesigned (scale bar is 400 μm).

In addition to dynamically exhibiting two-dimensional self-assembly of hexagonal silicon microplatelets at water-air interface of suspended water droplets, we also investigated two-dimensional self-assembly of microplatelets inside the water with much larger area and well-ordered predesigned patterns with much higher external energy input. In the proposed method, all faces of microplatelets were functionalized with Trichloro(1H,1H,2H,2H-heptadecafluorodecyl)silane; and lauryl methacrylate (which is a water insoluble liquid with low volatility) was deposited onto these functionalized surfaces. In this case, much higher kinetic energy in the form of ultrasonic waves were applied to the microplatelets in order to promote elementary building blocks to self-assemble into well-ordered structures (Fig 4a). The process involved in the proposed self-assembly method consisted of simply inserting the glass tube into ultrasonic bath (40 kHz operating frequency) without touching the bottom and the walls; and holding it in position, keeping the meniscus of glass tube higher than the water level of ultrasonic bath while self-assembly was occurring (Fig 4b). After the self-assembly process was accomplished, the glass tube with organized pattern consisting of microplatelets in the water environment could be observed by using stereomicroscope (Fig 4c). During the proposed self-assembling process, these microplatelets with surfaces that were hydrophobically modified and water insoluble liquid coated were suspended in water while being agitated by the energy of the ultrasonic waves. Large amounts of collisions happened between microplatelets, as well as between the microplatelets and the glass tube, resulting in the contacts and coalescences of the water insoluble liquid on those surfaces. The surface tension that resulted from the tendency of minimizing interfacial surface energy in this water environment held the insoluble liquid in the porous unordered aggregates with numerous gaps, preventing it from ascending to the top of the glass tube though its density is a bit lower than deionized water. The microplatelets that existed near the settled insoluble liquid tended to adhere to this liquid with their faces. For the microfabricated hexagonal microplatelets herein, the area of top and bottom faces was over 4 times that of the side faces. This led to the coalescence of the water-insoluble liquid, minimizing the interfacial area and causing the adhesion of top or bottom faces of microplatelets to the bottom concave surface of glass tube (Fig 4d).

Traditionally, water-air interface has been an ideal place for self-assembly demonstrated by previous researchers [3, 11, 41, 43], due to the fact that surface tension of water can hold enough particles and the hydrophobic nature can drive them to the surface; while, in solution, the driving force to reduce the surface area of the hydrophobic faces is too large for ordered assembly, resulting in haphazard aggregates formed by building blocks. In this study, however, we demonstrate that for mesoscale self-assembly of microplatelets, well-ordered two-dimensional aggregation can happen inside water (specifically, at the bottom concave surface of the glass tube in this case). Fig 4e demonstrates that hexagonal microplatelets aggregated into a dense and well-packed two-dimensional structure attaching to the interior surface of the glass tube for the minimization of their interfacial surface energy. In this case, the bottom interior surface of the glass tube provided a large smooth area for the microplatelets to attach to; this constraint of free movement of the microplatelets became a favourable factor—a substrate for the formation of the tessellated two-dimensional self-assembly. The area of this substrate was large enough that many microplatelets at the bottom of the glass tube can firmly attach to the substrate with their top or bottom faces. Meanwhile, the side faces of the microplatelets coalesced forming tessellated structures. In contrast to self-assembly at water-air interface, we achieved highly ordered two-dimensional self-assembly of microplatelets in water occurred with area more than 10 mm^2^. Although some parts of the self-assemblies may inevitably grow along both the in-plane and out-of-plane directions resulting in patterns with a few repeated layers, the overall trend of in-plane directional growth was significantly dominant considering the large tessellated area. Besides, these two-dimensional self-assemblies were stable enough that the 40 kHz ultrasonic wave was not able to break them apart. It can be seen from Fig 4f that even for the area where defect of self-assembly exists, the built structure was still precisely organized with a tendency to minimize defects and form a predesigned pattern. These results may open a potential application of surface coating technology by using microplatelets through autonomous self-assembly to mimic the nature’s design rule of creating hierarchically and precisely ordered two-dimensional patterns.

### Bio-inspired structure built by quick three-dimensional self-assembly

**Fig 5:**
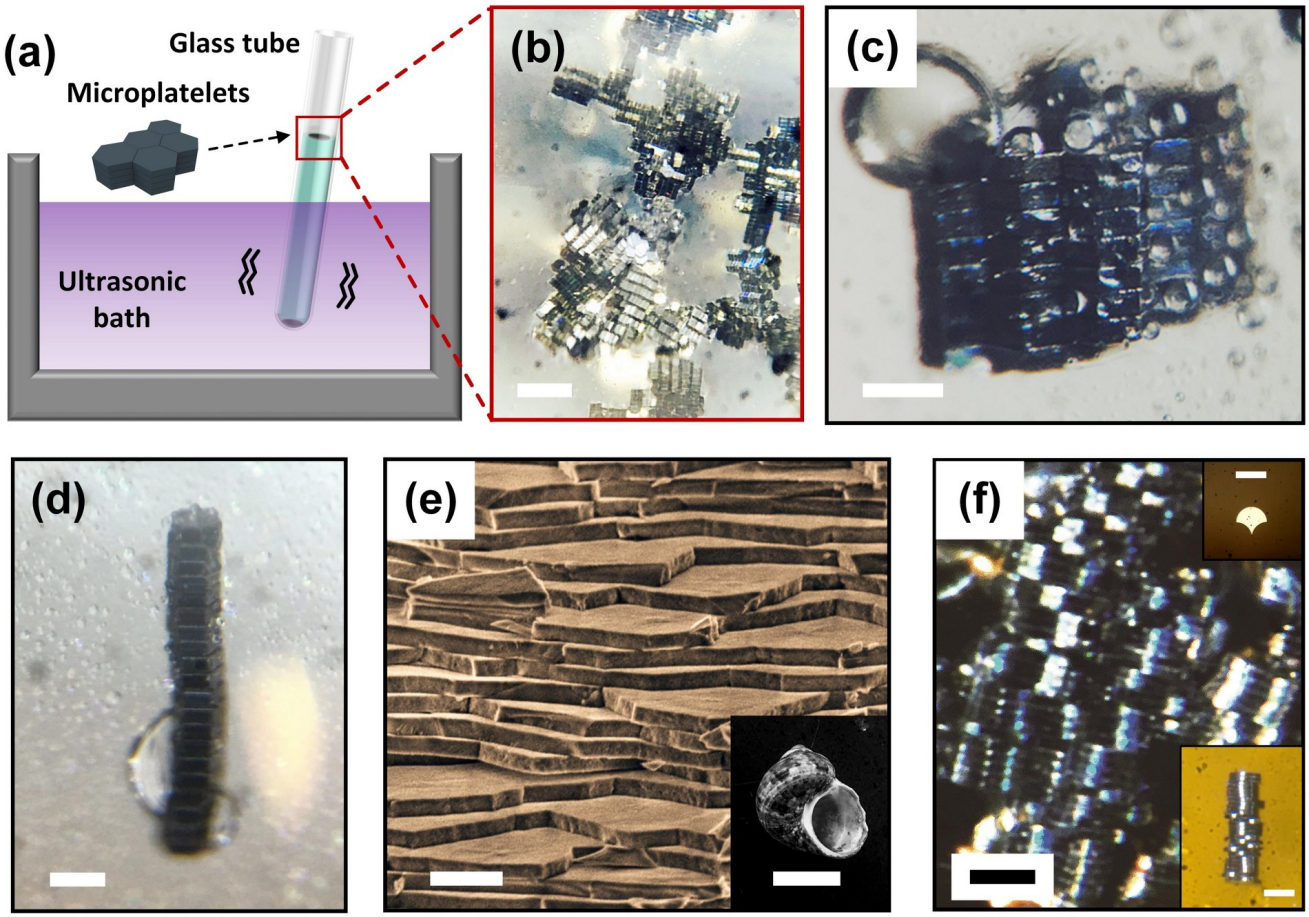
Formation of three-dimensional well-ordered self-assembly of microplatelets at the water-air interface of the glass tube. (a) Schematic illustration showing the self-assembled structures appeared at the water-air interface of glass tube in comparison with two-dimensional self-assembly that appeared at the bottom concave. (b-d) Optical images of (b) organized and stable three-dimensional multi-layered self-assembly that interconnected each other forming aggregates with size of several millimeters (scale bar is 500 μm); (c) crystalline structure which consisted of multi-layered stacks that extended in the hexagonal plane and in the direction perpendicular to it (scale bar is 100 μm); (d) long bar was well-ordered as a stacked structure without misalignment (scale bar is 100 μm). (e) SEM image of the nacre from mollusk shells (inset; scale bar is 1 cm) made of microscopic tablets of calcium carbonate (scale bar is 5 μm). (f) Optical image of biomimetic artificial nacre that was self-assembled by using fish-scale shape microplatelets (inset) as building blocks (scale bar is 100 μm).

In addition to two-dimensional self-assembly of microplatelets by using ultrasonic waves, the formation of three-dimensional ordered structure made up of microplatelets through self-assembly happened at the water-air interface of the glass tube. Strong agitation of water due to the energy of ultrasonic waves were capable of driving microplatelets to the water-air interface along with the bubbles created from vibration; and water-air interface was an ideal place where surface tension was strong enough to be able to hold abundant microplatelets for completing the self-assembling process (Fig 5a). The bubbles generated at the bottom of the glass tube constantly moved upwards to the meniscus. The movement and the high-frequency agitation of the bubbles drove the microplatelets around them to ascend from the bottom of the glass tube to the water-air interface. Thus, an increasing number of microplatelets reached the water-air interface where they can move spatially and freely, resulting in the occurrence of three-dimensional self-assembly. High-frequency vibration of the water in a glass tube due to ultrasonic waves significantly increased the chances of the collisions between a large number of building blocks (~2×10^5^); these collisions induced the coalescences between water-insoluble liquid on the eight faces of the hexagonal microplatelets, enabling the thin liquid films between adjacent faces to merge. The primary purpose of mechanical energy input was to provide enough agitation to the building blocks in the water environment and at the water-air interface. This energy input not only led to the adhesion of the microplatelets but also made the faces with the same geometry match each other to reduce overall solvation free energy. Over this course, a stable and well-ordered three-dimensional multi-layered self-assembled structure eventually formed at the air-water interface in 30 minutes in contrast to 1–3 days reported from the classical method of rotating cuvette [21]. It can be seen from Fig 5b that the self-assembled structure consisted of small multi-layered stacked crystalline structures that interconnected each other to form a larger aggregation several millimeters in size. Although a large number of building blocks with much greater destructive inertia force participated in the mesoscale self-assembly process, the aggregates exhibited a clear tendency towards the formation of the patterned structures. The faces of highly hydrophobic building blocks had low surface energy and in water were driven strongly to assemble with deep energy minima. Sonication provided sufficient energy to overcome these substantial energy barriers and reach a structure substantially similar to the global minimum. Fig 5c demonstrates 100 μm hexagonal microplatelets self-assembled into crystalline structure which consisted of multi-layered stacks that extended in the hexagonal plane and in the direction perpendicular to it. This three-dimensional mesoscale self-assembly of microplatelets were internally well-ordered and externally like a crystalline hexagonal lattice. The crystalline structures tended to extend further along the axis perpendicular to the hexagonal plane than in the in-plane directions, and there is no observable spatial orientation of the structures with respect to the water surface. It can be seen from Fig 5d that the long bar can be well-ordered as a multilayer structure with no top or bottom face of hexagonal microplatelets misaligned with respect to the adjacent ones. The fact that monolayer self-assembly was not found and the existence of accurately self-assembled long bars indicates that the upper and bottom faces of microplatelets with much larger area than side faces had significantly higher chances to precisely align and match, even though all faces of microplatelets were identically functionalized.

It is intriguing that many structural materials (nacre, bone or enamel, etc.) found in nature incorporate a large fraction of mineral building blocks to generate the stiffness and hardness required for their function, including structural support, protection and mastication, etc. [42]. These materials are tough, durable, damage tolerant and can even produce quasi-ductile behaviour. For instance, nacre from mollusc shells is made of microscopic tablets of calcium carbonate as can be seen from the scanning electron microscopy (SEM) image (Fig 5e). Cracks in nacre propagate along the interfaces, circumventing the microscopic tablets and generating tortuous path, which dissipate more energy, making nacre 3,000 times more fracture resistant than a single crystal of the pure mineral [44, 45], and it can undergo up to 1% tensile strain before failure; this is an exceptional amount of deformation compared to monolithic ceramics [42]. Today’s most advanced composites has yet to achieve the order and sophisticated hierarchy of hybrid material built up by living organisms in nature [41], which motivates scientists who have long sought to create strong and stiff layered structure to mimic artificial nacre. Herein, we demonstrate an effective route to a biomimetic artificial nacre that was self-assembled by using fish-scale shape microplatelets with 10 μm thickness and 100 μm width as elementary building blocks (Fig 5f). Similar to the hexagonal shape, in principle, the fish-scale shape has the property to interlock in one orientation but can rotate freely until sufficiently constrained by the neighbors. In this case, since the thickness of fish-scale shape microplatelets was only one third of that of hexagonal microplatelets, the fish-scale shape microplatelets had a stronger tendency to assemble with their top or bottom faces. It can be seen that the developed bio-inspired three-dimensional self-assembled structure serves as a structural analogue to the natural nacre with stiff layered structure. Compared to existing strategies [46–52], our proposed approach constructed the bio-inspired complex structural materials in an autonomously self-assembled manner without human intervention, and the whole building process was accomplished in a short amount of time (less than 30 minutes). This result demonstrates promise that the proposed method of three-dimensional mesoscale self-assembly will not only be a feasible but likely easy strategy to construct artificial nacre or other complex structural materials in nature. More importantly, this self-assembly strategy exploited nature’s design rule of creating hierarchically and precisely ordered structure through a bottom-up approach.

## Conclusions

In this article, we report the methods and progress of fast two- and three-dimensional well-ordered mesoscale self-assembly of microfabricated microplatelets (100 μm in size). The research aims to open the way towards higher order structures with length scales and architectural complexity that one day might approach many structure materials found in nature, and novel two-dimensional surface coating technology with autonomous organization of building blocks. The exploration and demonstration in this article could lay the foundations for potential advances in our ability to control the material world and offers the prospect of highly efficient, large volume and highly stable bottom-up manufacturing process of functional material.

## Supporting information

S1 File (DOCX)

S1 Movie Demonstration of real-time monolayer self-assembly of hexagonal microplatelets in droplets. (MOV)
