# Supplementary material for: Quick self-assembly of bio-inspired multi-dimensional well-ordered structures induced by ultrasonic wave energy
Source: PLoS One. 2021 Feb 24;16(2):e0246453. doi: 10.1371/journal.pone.0246453 (PMC7904215; doi:10.1371/journal.pone.0246453)
Supplement: S1 File — (DOCX) [file pone.0246453.s001.docx]

Quick self-assembly of bio-inspired multi-dimensional well-ordered structures induced by ultrasonic wave energy

Connor Murphy^1^, Yunqi Cao^2^, Nelson Sepúlveda^3^, Wei Li^1*^

^1^ Department of Mechanical Engineering, University of Vermont, Burlington, Vermont, United States of America

^2^ Ming Hsieh Department of Electrical and Computer Engineering, University of Southern California, Los Angeles, California, United States of America

^3^ Department of Electrical and Computer Engineering, Michigan State University, East Lansing, Michigan, United States of America

^*^ Corresponding author

E-mail: wei.li@uvm.edu

Supporting information

S1 Fig. Aggregation of microplatelets in the water environment prior to the input of ultrasonic wave energy (scale bar is 200 µm).

S2 Fig. Designs of (a) hexagonal microplatelets and (b) fish-scale shape microplatelets when tessellated in two dimensions.

S1 Movie. Demonstration of real-time monolayer self-assembly of hexagonal microplatelets in droplets. (Supporting movie S1.mov)
